# Exploring the impact of age of onset on stereoacuity in patients with myasthenia gravis

**DOI:** 10.3389/fopht.2025.1701092

**Published:** 2026-01-12

**Authors:** Chen-Yun Cheng, Hou-Chang Chiu, Chi-Feng Hung, Jiann-Horng Yeh

**Affiliations:** 1 Institute of Public Health, School of Medicine, National Yang Ming Chiao Tung University (NYCU), Taipei, Taiwan; 2 Neurology Department, Taipei Medical University-Shuang Ho Hospital, New Taipei, Taiwan; 3 Neurology Department, Shin Kong Wu Ho-Su Memorial Hospital, Taipei, Taiwan; 4 School of Medicine, Fu Jen Catholic University, New Taipei, Taiwan

**Keywords:** *Myasthenia gravis*, stereoacuity, age of onset, diplopia, extraocular movement, ptosis

## Abstract

**Introduction:**

Myasthenia gravis (MG) is an autoimmune disorder that can result in fluctuating muscle strength and various ocular manifestations. Common ocular signs and symptoms in MG patients include ptosis, limited eye movement, and diplopia.

**Purpose:**

This study aimed to investigate the association between the clinical characteristics of MG, specifically the age of onset, and stereoacuity in MG patients.

**Design:**

This was a cross-sectional study that enrolled 150 MG patients.

**Methods:**

Stereoacuity was assessed using the Butterfly Stereo Acuity Test. Main analysis was conducted using multinomial logistic regression to explore the relative risk associated with different levels of stereoacuity.

**Results:**

This study included 150 MG patients: 58 (38.7%) with normal stereoacuity, 80 (53.3%) with weak stereoacuity, and 12 (8%) unable to identify any stereo chart. Patients with MG onset before age 7 (n = 15) had a significantly higher risk of being unable to identify any stereo chart [relative risk ratio (RRR) = 14.73; p = 0.03]. Among ocular manifestations, ptosis (RRR = 2.33; p < 0.05) and diplopia (RRR = 2.26; p < 0.05) were associated with weak stereoacuity, whereas extraocular movement (EOM) disorder was significantly associated with being unable to identify any stereo chart (RRR = 10.57; p < 0.01).

**Conclusion:**

Findings suggest that an early onset of MG may contribute to impaired stereoacuity. For patients diagnosed with MG before the age of 7, it is advisable to ensure optimal management of MG itself. Future studies should explore the potential long-term implications of early-onset MG on binocular vision, ophthalmoplegia, and the impact of ocular signs and symptoms on patients’ quality of life.

## Introduction

Myasthenia gravis (MG) is an autoimmune disorder that disrupts neuromuscular transmission, leading to fluctuating muscle strength and increased fatigue (1). Ocular manifestations, including ptosis, limited eye movement, and diplopia, are prominent features in both ocular myasthenia gravis (OMG) and generalized myasthenia gravis (GMG) (2). In Taiwan, the incidence of MG is significantly higher in the elderly (3) and, following the worldwide epidemiological tendencies, with a steady rise in prevalence over time (4).

Recent advancements in medical care and pharmaceuticals have made the progression of MG largely manageable. However, despite these improvements, patients often continue to complain about their quality of life (5, 6). Clinically, both OMG and GMG patients frequently report a loss of spatial awareness; difficulties in reading, navigating stairs, and driving; and even incidents of falling, along with increasing instances of eye strain and related issues (7). As stereopsis represents the highest function in binocularity, this study assessed binocular function using stereoacuity (8). Therefore, this study aimed to explore the potential risk factors of impaired binocular vision in MG by screening the stereoacuity of MG patients, with a particular focus on the age of disease onset and its impact on stereoacuity. The study will focus on the age of MG onset (pre-specified an age cut-off of 7 years to evaluate the potential impact of MG onset during the pre-school stage on stereoacuity development) and associated ocular signs and symptoms (s/s) and will extensively investigate the association between MG and thyroid disorders, as these conditions frequently coexist and may exacerbate ocular symptoms (6, 9, 10).

## Material and methods

### Study setting and participants

This cross-sectional study enrolled 150 MG patients who were consecutively seen at the Neurology Department of Shin Kong Wu Ho-Su Memorial Hospital (SKH), Taiwan, between April and September 2017. Eligible participants had a confirmed MG diagnosis by a neurologist, had no other ocular diseases, and provided written informed consent approved by the Institutional Review Board. To ensure the exclusion of non-MG-related ocular conditions, patients were asked about any history of ocular disease before consent, and their medical records were reviewed for verification. Because thyroid dysfunction, another autoimmune condition, commonly coexists with MG, patients with thyroid dysfunction were included. One patient with a history of Graves’ disease was retained due to the small number of thyroid-related cases and the documented stability of ocular symptoms. MG diagnosis was confirmed by at least one of the following: presence of serum antibodies, results from repetitive nerve stimulation, or findings from single-fiber EMG. All participants were clinically stable for at least 1 month before enrollment.

### Outcome ascertainment

We measured stereoacuity using the *Butterfly Stereo Acuity Test with Lea Symbols^®^*. Patients were required to have the best-corrected prescription for near and position themselves 16 inches away from the testing book. The test book presented images with matched luminance levels and crossed disparities ranging from 2,500 to 20 arc-seconds. We defined normal stereoacuity as 40 arc-seconds or better. If a participant was unable to differentiate any stereoscopic chart in the Stereo Acuity Test, we recorded the result as “fail to identify”. Stereoacuity worse than 40 arc-seconds but within the measurable range of 40 to 2,500 arc-seconds was classified as weak stereoacuity.

### Evaluating ocular signs and symptoms of MG

#### Ptosis assessment

Ptosis was classified into three levels based on the extent of eyelid coverage over the pupil and was measured using a pupillary distance ruler. Mild ptosis was defined as approximately 2 mm of eyelid drooping or drooping that partially covered the iris without reaching the center of the pupil. For unilateral ptosis, the degree of drooping was determined by comparing the affected eye with the contralateral normal eye. For bilateral ptosis, mild severity was assigned when both eyelids covered the iris by approximately 2 mm but did not reach the center of the pupil. Moderate ptosis was defined as eyelid coverage reaching the center of the pupil in either eye. Severe ptosis was characterized by the corneal light reflex being obscured in either eye.

#### Evaluation of EOMs and diplopia

The evaluation of extraocular movement (EOM) function and diplopia was conducted using a non-accommodative target at 50 cm in nine directions. EOM disorder was defined as any observed movement limitation, assessed both subjectively and objectively. Subjectively, patients reported difficulty moving their eyes and its frequency over the past 3 months. Objectively, eye movements were evaluated for coordination and conjugacy, repeated three times. Based on movement difficulty and frequency, EOM function was classified into four levels but analyzed as two categories: “normal EOMs” (normal/mild) and “EOM disorder” (moderate/severe). Diplopia was assessed during EOM testing by asking patients if they saw double images and their relative position, repeated three times. Its severity was also categorized into “normal” (normal/mild) and “abnormal” (moderate/severe) for analysis of its relationship with stereoacuity. (**Supplementary Tables S3**, **S4**).

### Covariates

This study retrospectively reviewed patients’ basic information, including onset, MG subtype according to the muscle groups involved, the duration of MG course, and the presence of thyroid-associated diseases. The classification of MG into generalized and ocular subtypes followed clinical standards and was based on the Osserman grade and the MGFA classification (11).

### Statistical analysis

Analysis was performed using Stata 18.0. Continuous variables were reported as mean ± SD, and categorical variables as number (percentage). Group comparisons used t-tests and chi-square tests. Multinomial logistic regression assessed stereoacuity, with univariate/multivariable models analyzing MG onset age and stereoacuity levels, reporting relative risk ratios (RRRs) along with 95% CIs.

## Results

### Distribution and clinical characteristics of the study group

A total of 150 MG cases (male/female ratio, 0.56) were included, with a mean age of 48.5 years (range, 10.5–80.7). Of these, 58 (38.7%) demonstrated normal stereoacuity, 80 (53.3%) had weak stereoacuity, and 12 (8%) were unable to identify any stereo chart. Patients with absent stereopsis had a longer disease duration (median, 20.5 years; p < 0.001) and a higher prevalence of thyroid-associated disease (25%; p < 0.01) compared with the other groups. In addition, ocular manifestations—including ptosis, EOM disorder, and diplopia—were significantly more prevalent with worsening stereoacuity. Conversely, patients with normal stereoacuity showed the opposite pattern; they tended to have a shorter disease duration (median, 10.3 years; p < 0.001) and fewer ocular signs and symptoms (**Table 1**).

**Table 1:** Clinical characteristics in patients with myasthenia gravis.

Clinical characteristics	Total N = 150	Stereoacuity			p-Value
		Normal n = 58 (38.7%)	Weak n = 80 (53.3%)	Fail to identify any stereo chart n = 12 (8%)	
Age (years)^a^					0.365
	48.5 ± 16.5	50.4 ± 16.2	46.7 ± 17.0	51.0 ± 14.7	
Gender^b^					0.591
Male	54 (36.0%)	18 (31.0%)	31 (38.8%)	5 (41.7%)	
MG type^b^					0.465
Ocular form	75 (50%)	29 (50%)	38 (47.5%)	8 (66.7%)	
Generalized form	75 (50%)	29 (50%)	42 (52.5%)	4 (33.3%)	
Age of MG onset (years)^a^					0.237
	36.0 ± 18.7	36.9 ± 17.7	36.7 ± 18.8	27.3 ± 21.5	
Disease duration (years)^c^					**<0.001**
	9.6 (3.2, 17.6)	10.3 (5.4, 18.6)	6.3 (2.0, 14.3)	20.5 (15.2, 35.4)	
Ocular signs and symptoms^b^					
Ptosis	57 (38.0%)	13 (22.4%)	37 (46.3%)	7 (58.3%)	**0.006**
EOM disorder	28 (18.7%)	4 (6.9%)	17 (21.3%)	7 (58.3%)	**<0.001**
Diplopia	68 (45.3%)	17 (29.3%)	43 (53.8%)	8 (6.7%)	**0.005**
Comorbidities with AITD^b^					
	9 (6%)	1 (1.72%)	5 (6.3%)	3 (25%)	**0.008**

Continuous variable without a normal distribution was presented as median (IQR). Significance was set as p < 0.05 and marked in bold. Normal stereoacuity was defined as the ability to discern stereoscopic targets with a disparity of 40 arc-seconds or less. The maximum disparity angle of the stereoscopic target in this test was 2,500 arc-seconds.

MG, myasthenia gravis; EOM, extraocular movement; AITD, autoimmune thyroid disorder, including hyperthyroidism and Graves’ disease.

a ANOVA was used to estimate the relationship between the variables.

b Chi-square test was used to estimate the relationship between the variables.

c Kruskal–Wallis test was used to perform the analysis.

### Association of the age of MG onset with stereoacuity

To explore whether the effect of MG onset on stereoacuity differed across developmental and adult age ranges, multiple age cut-offs (7, 8, 20, and 50 years) were examined. Although the 7-year threshold was prespecified to capture the potential influence of MG onset during the pre-school stage on stereoacuity development, additional cut-offs were evaluated to assess age-related patterns across the broader disease population. No significant risk difference was found when comparing the onset before and after 20 years. However, patients with onset before 7 years of age had a significantly higher risk ratio (RRR = 14.73; p = 0.03) of being unable to identify a stereo chart compared to those whose onset occurred at or after age 7 (**Table 2**).

**Table 2:** Relative risk ratio of onset age by multinomial logistic regression analysis for different levels of stereoacuity.

Independent variables	Dependent variable category			
	Weak stereoacuity		Fail to identify any stereo chart	
	(Ref. Normal stereoacuity)			
	Model 1^a^	Model 2^b^	Model 1^a^	Model 2^b^
	RRR (95% CIs)	RRR (95% CIs)	RRR (95% CIs)	RRR (95% CIs)
Onset age ≤ 7 (n = 15)	8.14 (1.01, 65.52)*	8.21 (0.95, 70.63)	28.50 (2.82, 287.9)**	14.73 (1.22, 177.34)*
Ptosis		2.65 (1.15, 6.13)*		2.15 (0.47, 9.87)
EOM disorder		1.54 (0.43, 5.44)		7.07 (1.24, 40.35)*
Diplopia		2.31 (1.08, 4.98)*		2.78 (0.64, 12.11)
MG type (OMG)		0.66 (0.31, 1.40)		1.29 (0.29, 5.71)
Onset age ≤ 8 (n = 16)	4 (0.84, 19.0)	4.03 (0.79, 20.61)	14 (2.20, 89.22)*	7.57 (0.97, 59.12)
Ptosis		2.62 (1.14, 6.03)*		2.13 (0.47, 9.73)
EOM disorder		1.63 (0.47, 5.72)		7.45 (1.32, 42.17)*
Diplopia		2.33 (1.09, 4.98)*		2.79 (0.64, 12.15)
MG type (OMG)		0.69 (0.33, 1.47)		1.37 (0.31, 5.96)
Onset age < 20 (n = 34)	0.87 (0.38, 1.98)	0.70 (0.28, 1.78)	2.47 (0.67, 9.10)	1.01 (0.21, 4.75)
Ptosis		2.45 (1.07, 5.59)*		1.94 (0.43, 8.64)
EOM disorder		2.31 (0.64, 8.31)		10.81 (1.83, 63.97)**
Diplopia		2.21 (1.04, 4.69)*		2.61 (0.61, 11.14)
MG type (OMG)		0.75 (0.36, 1.59)		1.67 (0.39, 7.05)
Onset age < 50 (n = 111)	0.98 (0.45, 2.12)	0.96 (0.42, 2.20)	1.05 (0.25, 4.39)	0.49 (0.09, 2.54)
Ptosis		2.50 (1.10, 5.70)*		1.92 (0.43, 8.57)
EOM disorder		2.03 (0.59, 6.97)		13.32 (2.30, 77.13)**
Diplopia		2.23 (1.05, 4.76)*		2.50 (0.59, 10.65)
MG type (OMG)		0.75 (0.36, 1.57)		1.94 (0.45, 8.47)

Significance was set as p < 0.05 and marked with an asterisk.

RRR, relative risk ratio; OMG, ocular myasthenia gravis; EOM, extraocular movement.

a Model 1: univariate model.

b Model 2: full-adjusted model, adjusted for ptosis, EOM disorder, diplopia, and MG type.

*p < 0.05; **p < 0.01.

### The predicted relative risk ratio of MG-associated ocular s/s for different levels of stereoacuity

Multinomial logistic regression was conducted to investigate the association between the individual effects of each ocular s/s and the different levels of stereoacuity. **Table 3** presents the RRR by comparing each ocular s/s to patients who did not exhibit ocular s/s across different levels of impaired stereoacuity relative to normal stereoacuity. Compared to patients with normal stereoacuity, those manifesting ptosis and diplopia had RRRs of 2.33 (p = 0.04) and 2.26 (p = 0.03), respectively, for weak stereoacuity. However, the RRR for EOM disorder was 10.57 (p = 0.01) for the development of more severe stereoacuity dysfunction, as compared to those with normal stereoacuity (**Table 3**).

**Table 3:** Relative risk ratio of MG-associated ocular signs and symptoms based on multinomial logistic regression analysis for different levels of stereoacuity.

Independent variables	Dependent variable category			
	Weak stereoacuity		Fail to identify any stereo chart	
	(Ref. Normal stereoacuity)			
	Model 1^a^	Model 2^b^	Model 1^a^	Model 2^b^
	RRR (95% CIs)	RRR (95% CIs)	RRR (95% CIs)	RRR (95% CIs)
Ptosis (n = 57)	2.98 (1.40, 6.35)**	2.33 (1.05, 5.17)*	4.85 (1.32, 17.84)*	2.19 (0.51, 9.38)
EOM disorder (n = 28)	3.64 (1.16, 11.48)*	2.05 (0.60, 6.97)	18.9 (4.08, 87.50)**	10.57 (2.01, 55.63)*
Diplopia (n = 68)	2.80 (1.37, 5.74)**	2.26 (1.07, 4.78)*	4.82 (1.28, 18.18)*	2.50 (0.59, 10.59)

Significance was set as p < 0.05 and marked with an asterisk.

RRR, relative risk ratio; OMG, ocular myasthenia gravis; EOM, extraocular movement.

a Model 1: univariate model.

b Model 2: full-adjusted model, adjusted for the other ocular signs and symptoms (s/s) (ptosis, EOM disorder, and diplopia).

*p < 0.05; **p < 0.01.

## Discussion

This study investigated stereopsis function in MG patients, revealing a higher risk of abnormal stereoacuity in those with onset before age 7. Ptosis and diplopia were linked to weak stereoacuity, while EOM disorder was strongly associated with stereoacuity loss. Patients who were unable to identify any stereopsis images had a longer disease duration and a higher prevalence of autoimmune thyroid disorder (AITD).

The cross-sectional design limits causal interpretation, as some patients may have had impaired stereoacuity before MG diagnosis (e.g., childhood strabismus). Despite ensuring binocular near visual acuity (VA) (20/30) before testing, functional vision variations could affect results (12). Differences in accommodation, convergence, and age-related stereoacuity sensitivity may introduce measurement errors (13). Additionally, there are inherent limitations in evaluating MG patients whose ocular manifestations fluctuate, as single-time clinical assessments may not fully capture disease variability and could therefore influence the accuracy of stereopsis testing. Future studies should explore how evaluation timing influences outcomes.

Moreover, the Butterfly Stereo Acuity Test does not strictly distinguish true binocular stereopsis from monocular depth cues. Thus, patients with impaired binocular fusion may still achieve measurable stereoacuity scores based on monocular cues such as relative size, shadowing, or motion parallax. This limitation may lead to an overestimation of stereoacuity in some patients. Future studies may consider incorporating additional binocularity assessments (such as the Worth Four Dot Test) to obtain a more comprehensive evaluation of binocular status in MG patients.

This study set the onset age cut-offs at 7 and 20 years, considering emmetropization completion by age 6–7 years (14, 15) and refractive error stabilization by 16–21 years (16). To validate the 7-year cut-off, it was extended to 8 years. MG onset before 8 years was associated with a 14-fold higher risk of stereoacuity loss in the crude model, although this decreased after adjusting for confounders. Comparing early-onset (<50 years) and late-onset (≥50 years) MG, no increased risk of abnormal stereoacuity was found. These findings suggest and support that MG onset before emmetropization increases the risk of stereoacuity impairment, highlighting the long-term impact of early visual development disruption (17, 18).

During the exploration of MG onset earlier than 6 years, the findings not only predicted abnormal stereoacuity but also indicated that all 12 patients diagnosed at or before 6 years of age experienced a certain level of stereoacuity dysfunction (**Supplementary Table S1**). Despite a limited sample size, these findings suggest that young age is critical for the development of binocular vision, as indicated in previous studies (14, 15). Thus, if MG is clinically diagnosed in a patient before being of pre-school age, a consultation with an ophthalmologist or optometrist is advised.

Misalignment of the visual axes can lead to diplopia. This study found a strong association between EOM disorder and diplopia (**Supplementary Table S2**), with 75% of patients with EOM disorders experiencing diplopia. However, 25% reported no double vision, possibly due to neural suppression of the deviating eye. Further research is needed to determine whether inadequate diplopia management in MG contributes to this suppression.

Given the rarity of MG, the study included all eligible patients, providing a meaningful sample for exploratory analysis of binocular visual function while acknowledging the limited size of the smallest outcome category. The survey-based design and single-site sample limit generalizability. However, as SKH is Taiwan’s primary MG center, findings likely reflect the northern Taiwan MG population. Future studies should adopt more robust study designs, incorporating longer observation periods, larger sample sizes, and broader potential confounders to explore the impact of onset age, EOM capabilities, and diplopia severity on stereoacuity and patients’ quality of life, particularly in those with early onset.

## Data Availability

The original contributions presented in the study are included in the article/**Supplementary Material**. Further inquiries can be directed to the corresponding author.
