# Supplementary material for: Exploring the impact of age of onset on stereoacuity in patients with myasthenia gravis
Source: Front Ophthalmol (Lausanne). 2026 Jan 12;5:1701092. doi: 10.3389/fopht.2025.1701092 (PMC12832507; doi:10.3389/fopht.2025.1701092)
Supplement: Supplementary file 1 [file DataSheet1.docx]

**Exploring the Impact of Age of Onset on Stereoacuity in Patients with Myasthenia Gravis**

**Table Captions**

There are four tables described in the supplementary material. Supplemental table 1 depicts the correlation between the age of MG onset younger than 6 years and different levels of stereoacuity. Supplemental Table 2 illustrates the correlation between extraocular movement disorder and diplopia. Supplemental Table 3 and 4 explain the definitions and classifications of MG-associated ocular manifestations, specifically the severity levels of EOMs disorder and diplopia occurrence, respectively.

**Supplemental Table 1 Correlation between age of MG onset younger than 6 years and different level of stereoacuity.**

|  | **Stereoacuity** | | | Total | p-value |
| --- | --- | --- | --- | --- | --- |
|  | Normal | Weak | Fail to identify any stereo chart |  |  |
| **Age of onset** |  |  |  |  |  |
| $\leq$ 6 yrs | 0 | 9 | 3 | 12 |  |
| $>$ 6 yrs | 58 | 71 | 9 | 138 |  |
| Total | 58 | 80 | 12 | 150 | 0.004 |

^a^Chi-square test was used to estimate the relationship between the variables.

**Supplemental Table 2 Correlation between extraocular movement disorder and diplopia.**

|  | **Diplopia** | | Total | p-value |
| --- | --- | --- | --- | --- |
|  | without | with |  |  |
| **EOM disorder** |  |  |  |  |
| without | 75 | 47 | 122 |  |
| with | 7 | 21 | 28 |  |
| Total | 82 | 68 |  | <0.001 |

^a^Chi-square test was used to estimate the relationship between the variables.

**Supplemental Table 3 The definition of extraocular movement (EOMs) disorder.**

| **EOMs disorders** | | Frequency | | |
| --- | --- | --- | --- | --- |
|  |  | none | Sometimes | Always |
| difficulty level in gazing at the 9 directions | none | ***normal*** | ***normal*** | ***normal*** |
|  | single direction in the horizontal plane | ***normal*** | ***mild*** | ***moderate*** |
|  | movement difficulty in more than two directions | ***normal*** | ***moderate*** | ***severe*** |

**Supplemental Table 4 The definition of diplopia severity.**

| **Severity of diplopia** | | Occurrence frequency | | | |
| --- | --- | --- | --- | --- | --- |
|  |  | none | Occasionally | Sometimes | Always or persistent |
| Occurrence position | none | ***normal*** | ***normal*** | ***normal*** | ***normal*** |
|  | Non-primary position | ***normal*** | ***mild*** | ***moderate*** | ***severe*** |
|  | Primary position | ***normal*** | ***moderate*** | ***severe*** | ***severe*** |

occurring occasionally when tired, i.e., occurring occasionally and for a short duration

occurring sometimes when tired, i.e., with diplopia lasting for a period and requiring rest to alleviate
